# A review of the Japanese species of the family Tischeriidae (Lepidoptera)

**DOI:** 10.3897/zookeys.601.7782

**Published:** 2016-06-29

**Authors:** Shigeki Kobayashi, Hiroaki Sato, Nagao Hirano, Kazutaka Yamada, Toshiya Hirowatari

**Affiliations:** 1 Entomological Laboratory, Graduate School of Life & Environmental Sciences, Osaka Prefecture University, Sakai, Osaka, 599-8531 Japan; 2 Department of Biological Sciences, Faculty of Science, Nara Women’s University, Kitauoya-Nishimachi, Nara, 630-8506 Japan; 3 9955-3, Shimojima, Hata, Matsumoto, Nagano, 390-1401 Japan; 4 Tokushima Prefectural Museum, Bunka-no-Mori Park, Mukôterayama, Hachiman-chô, Tokushima, 770-8070 Japan; 5 Entomological Laboratory, Faculty of Agriculture, Kyushu University, 6-10-1 Hakozaki, Fukuoka, 812-8581 Japan

**Keywords:** Betula, Carpinus, Corylus sieboldiana, genitalia, leafminer, mine, Ostrya, taxonomy

## Abstract

This paper provides taxonomic and biological data on one new and one newly recorded species of *Coptotriche* Walsingham and one new and one newly recorded species of *Tischeria* Zeller from Japan. *Coptotriche symplocosella* Kobayashi & Hirowatari, **sp. n.** (host *Symplocos lucida*, Symplocaceae), and *Tischeria kumatai* Sato, Kobayashi & Hirowatari, **sp. n.** (host *Tilia japonica*, Malvaceae) are described. The pupal morphology of *Coptotriche symplocosella* is illustrated with scanning electron micrographs. *Coptotriche minuta* Diškus & Stonis, 2014 and *Tischeria relictana* Ermolaev, 1986 are newly recorded from Japan. The female, hostplants (*Carpinus*, *Corylus*, and *Ostrya* species), and immature stages of *Coptotriche minuta* and the adult features, female, and hostplants (*Betula* species) of *Tischeria relictana* are described with photographs and drawings for the first time. Mine types and characters of Japanese Tischeriidae are reviewed with photographs.

## Introduction

The Tischeriidae is a lepidopteran family comprising some of the smallest moths, with a wing expanse of only 5–11 mm. Tischeriid adults are rather similar to one another in appearance, with a brown or blackish gray vestiture. The family can be distinguished from other families by a frontal tuft projecting over a triangular face smoothly covered with scales, numerous, long and recurved, cilia-like sensilla trichodea (see Davis 1986 and van Nieukerken and Dop 1987) on the male antenna, in the male genitalia by a strongly narrowed phallus, usually bifurcate or with apical spines, and in the female genitalia with four to five pairs of apophyses (Puplesis and Diškus 2003). Puplesis and Diškus (2003) recognized three genera: *Tischeria* Zeller, 1839, *Coptotriche* Walsingham, 1890, and *Astrotischeria* Puplesis & Diškus, 2003. Until now, 115 tischeriid species have been described globally. Nearly eleven plant families have been reported as their hosts, among which Rosaceae, Fagaceae and Asteraceae are major groups (Puplesis and Diškus 2003; Stonis et al. 2014). Several species of Tischeriidae have recently been added to the Eastern Palaearctic fauna, including two species from China (Huang and Tan 2009) and two new species from the Russian Far East (Stonis et al. 2014).

In Japan, two genera and seven species of Tischeriidae have been described to date (Sato 2011; see checklist below). In addition, some unnamed species have also been collected (Oku 2003, Sato 2011). Among them, according to Sato (2011), two are unidentified *Tischeria* species associated with *Tilia* (Malvaceae) and *Betula* (Betulaceae) respectively, and two are unidentified *Coptotriche* species associated with *Carpinus* (Betulaceae) and *Quercus* (Fagaceae) respectively.

In this paper, we taxonomically review the Japanese species of the family Tischeriidae, resolving the identity of three of these unidentified species with descriptions of two new species and two newly recorded species. For the two *Coptotriche* species, larval and/or pupal stages are also described. Eight Japanese species were reared, and their mine types and characters are reviewed with photographs.

## Materials and methods

Adults were collected with light traps and leaves with mining larvae and cocoons were sampled from March to November in 2008 to 2015 in locations shown in Table 1. Adult specimens are preserved in the Osaka Prefecture University
(OPU) and Tokushima Prefectural Museum
(TKPM). Immatures in leaves were reared in plastic cups (420 ml: 129 mm in top diameter and 60 mm in depth) containing wet cotton at 20±5 ˚C under a photoperiod of 13–16L : 8–12D in the laboratory. In addition, specimens collected by Dr H. Kuroko in OPU, those collected by Dr T. Kumata in Hokkaido University Museum
(HUM), and collections of the third author (Hirano) were examined.

**Table 1.** Study sites of Tischeriidae species.

Locality	Prefecture	Island	Longitude and latitude	Altitude (m)	Figure number
Sai-ko, Fuji-Kawaguchiko	Yamanashi	Honshu	35°29'58"N, 138°39'32’’E	930	
Soni, Uda	Nara	Honshu	34°30'N, 136°07'E	400–1000	1A–E
Mt. Wasamata, Kamikitayama	Nara	Honshu	34°13'05’’N, 135°58'58"E	1150	
Mt. Kumoso, Kamiyama	Tokushima	Shikoku	33°54'43.4"N, 134°17'22.7"E	1123	Hirowatari et al. (2011): 1A, B
Adachi Park, Kokura	Fukuoka	Kyushu	33°51'56"N, 130°54'21"E	80–150	1F
Mikata, Tsushima Is.	Nagasaki	Kyushu	34°17'10"N, 129°16'20"E	20–30	1G–I

Photographs of leaf mines were taken primarily in the field using an OLYMPUS μ1060 digital camera. Some leafmines were scanned using an EPSON GT7400. Some pupae were dried and sputter-coated with a 60 : 40 mixture of gold-palladium for examination with a scanning electron microscope (SEM). SEM photographs were taken using HITACHI SU1510 with a lanthanum hexaboride (LaB6) source at an accelerating voltage of 15 kV. For preparation of the male and female genitalia, the abdomen was removed and boiled for 3–4 min in 10% aqueous KOH. They were stained with acetocarmine.

Terms for genitalia, in principle, follow Stonis et al. (2014). The term “prela” introduced by Braun (1972) is used to designate the two or three paired rod-like or plate-like apophyses that extend from the inner side of the 8^th^ and 9^th^ sternites. The term “antrum” is employed to indicate the strongly thickened, differently shaped walls of the vestibulum following Puplesis and Diškus (2003). Scientific names of plants follow the Missouri Botanical Garden *Tropicos* database (2015).

### A checklist of the Japanese species of the family Tischeriidae

I. Genus ***Tischeria*** Zeller, 1839

1. ***Tischeria naraensis*** Sato, 1993

Distribution: Japan: Honshu (Kinki region).

Hostplants: *Quercus acutissima* and *Quercus variabilis*, Fagaceae.

2. ***Tischeria quercifolia*** Kuroko, 1982

Distribution: Japan: Hokkaido, Honshu, Shikoku, Kyushu.

Hostplants: *Quercus acutissima*, *Quercus crispula*, *Quercus dentata*, and *Quercus serrata*, Fagaceae.

3. ***Tischeria decidua siorkionla*** Kozlov, 1986

Distribution: Japan: Hokkaido, Honshu, Kyushu (Tsushima Is.); the Russian Far East.

Hostplants: *Quercus acutissima*, *Quercus crispula*, *Quercus dentata*, *Quercus serrata*, and *Quercus variabilis*, Fagaceae.

4. ***Tischeria kumatai*** Sato, Kobayashi & Hirowatari, sp. n.

Distribution: Japan: Hokkaido, Honshu (Nagano).

Hostplants: *Tilia japonica*, Malvaceae.

5. ***Tischeria relictana*** Ermolaev, 1986

Distribution: Japan: Hokkaido, Honshu, Shikoku; the Russian Far East.

Hostplants: *Betula ermanii* and *Betula grossa*, Betulaceae.

II. Genus ***Coptotriche*** Walsingham, 1890

6. ***Coptotriche angusticollella*** (Duponchel, 1843)

Distribution: Japan: Hokkaido, Honshu; Europe; Tunisia; Turkey; Caucasus; Turkmenistan; South Korea; the Russian Far East.

Hostplants: *Rosa multiflora*, *Rosa wichuraiana*, *Rosa* spp., Rosaceae.

7. ***Coptotriche heinemanni*** (Wocke, 1871)

Distribution: Japan: Honshu, Shikoku, Kyushu; Europe; Tunisia; South Korea; Russia.

Hostplants: Agrimonia pilosa var. japonica, *Geum japonicum*, *Rubus crataegifolius*, *Rosa microphyllus*, *Rosa leucodermis* and Rubus palmatus var. palmatus, Rosaceae.

8. ***Coptotriche japoniella*** Puplesis & Diškus, 2003

Distribution: Japan; China.

Hostplants: *Eurya emarginata* and *Eurya japonica*, Theaceae.

9. ***Coptotriche szoecsi*** (Kasy, 1961)

Distribution: Japan*; Europe.

Hostplants: *Sanguisorba officinalis*, Rosaceae.

* Hokkaido: subsp. *szoecsi*; Honshu: subsp. *japonica* (Kuroko, 1982).

10. ***Coptotriche minuta*** Diškus & Stonis, 2014

Distribution: Japan: Honshu, Shikoku, Kyushu; the Russian Far East.

Hostplants: *Carpinus cordata*, *Carpinus japonica*, *Carpinus laxiflora*, *Carpinus tschonoskii*, *Corylus sieboldiana* and *Ostrya japonica*, Betulaceae.

11. ***Coptotriche symplocosella*** Kobayashi & Hirowatari, sp. n.

Distribution: Japan: Kyushu.

Hostplants: *Symplocos lucida*, Symplocaceae.

## Taxonomy

### Coptotriche minuta

Taxon classificationAnimaliaLepidopteraTischeriidae

Diškus & Stonis, 2014

Figs 2A–C , 3A–D , 7 , 8

Coptotriche minuta Diškus & Stonis, 2014: 143–144, figs 5–10.
Coptotriche sp.: Sato 2011: 128, 559, figs II-14.3A, B.

#### Type locality.

Russia: the Russian Far East (Primorskiy Territory).

**Material examined.** 38 (17♂ 17♀ 4 exs).

Host *Carpinus cordata*: 1♀, Oshirakawa, Azumi, Matsumoto, Nagano Pref., 29.vii.1990, N. Hirano leg., 7.vii.1990(larva), (genitalia slide no. OPU-SK568)

Host *Carpinus japonica*: 1♂ 1♀, Oshirakawa, Azumi, Matsumoto, Nagano Pref., 26&29.iv.1991, N. Hirano leg., 27.x.1990(larva), SK563, 564; [Soni, Uda, Nara Pref., S. Kobayashi leg.]: 1♀, Konagao, 18.vii.2012em., 14.vii.2012(larva); 1♀, Kameyama, Taroji, 23.vii.2011em., 24.vi.2011(larva). 3♂, Kabuto-dake climb point, 9&16.ix.2010em., 17.x.2010(larva), SK406.

Host *Carpinus laxiflora*, S. Kobayashi leg.: 1♂, Mitsuigatani, Konagao, Soni, Uda, Nara Pref., 16.ii.2011em., 14.ix.2011(larva), SK407; 1♂ 1♀, Ohshirakawa, Nagawa, Matsumoto, Nagano Pref., 7.viii.2012em., 3.viii.2012(larva).

Host *Carpinus tschonoskii*: 1♂, Kiso-Hukusima, Nagano Pref., 27.iv.1976em., T. Kumata leg., Rearing code: Kumata 1520, Genitalia slide no. HS-G54, deposited in HUM (Sato, 2011: fig.II-14.3A); 1 ex, Tawamine, Konagao, Soni, Uda, Nara Pref., 18.viii.2015em., S. Kobayashi leg., 15.viii.2015(pupa); Ehime Pref.: 3♂ 4♀, Matsuyama, 23.iv.1965, H. Kuroko leg.; 1♀, Nametoko nr. Uwazima, 1.v.1981em., T. Kumata leg., K2279, HS-G57 (HUM) (Sato, 2011: fig.II-14.3B); [Hikosan, Fukuoka Pref., H. Kuroko leg.]: 1♀ 1 ex, 2.v.&22.vii.1954; 2♀ 1 ex, 1&4.v.1957; 1♀, 10.viii.1957.

Host *Corylus sieboldiana*: 1♂, Saiko-nishi, Fuji-Kawaguchiko, Yamanashi Pref., 16.viii.2011em., S. Kobayashi leg., 6.viii.2011(larva), SK408. 1♀, Mt. Kuroiwa, Nagano Pref., 6.iv.1987em., H. Kuroko leg., 16.x.1986(larva).

Host *Ostrya japonica*: 1♂, Sapporo, Hokkaido, 21.vii.1959, T. Kumata leg.

Host unknown: 5♂ 2♀ 1 ex, Mt. Wasamata, Nishihara, Kamikitayama, Nara Pref., 23&24.viii.2011, collected by light trap (L.T.), T. Hirowatari, K. Ikeuchi, Y.-S. Bae & S. Kobayashi leg., SK570.

#### Diagnosis.

See original description.

#### Additional description.

**Adult** (Fig. 2A–C). Male and female. Wing expanse 6.6–8.8 mm in Japanese specimens, 8.6, 8.8 mm in hibernating generation, and 6.6–7.7 mm in summer and autumn generations. Forewing (Fig. 2A–C) pale to dark ocherous, especially blackish brown in overwintering form (Fig. 2C). Japanese specimens of summer and autumn generations have more distinctly grayish brown scales along the costal margin and apical part of the forewing than the type series collected in July and August.

**Figure 1.**
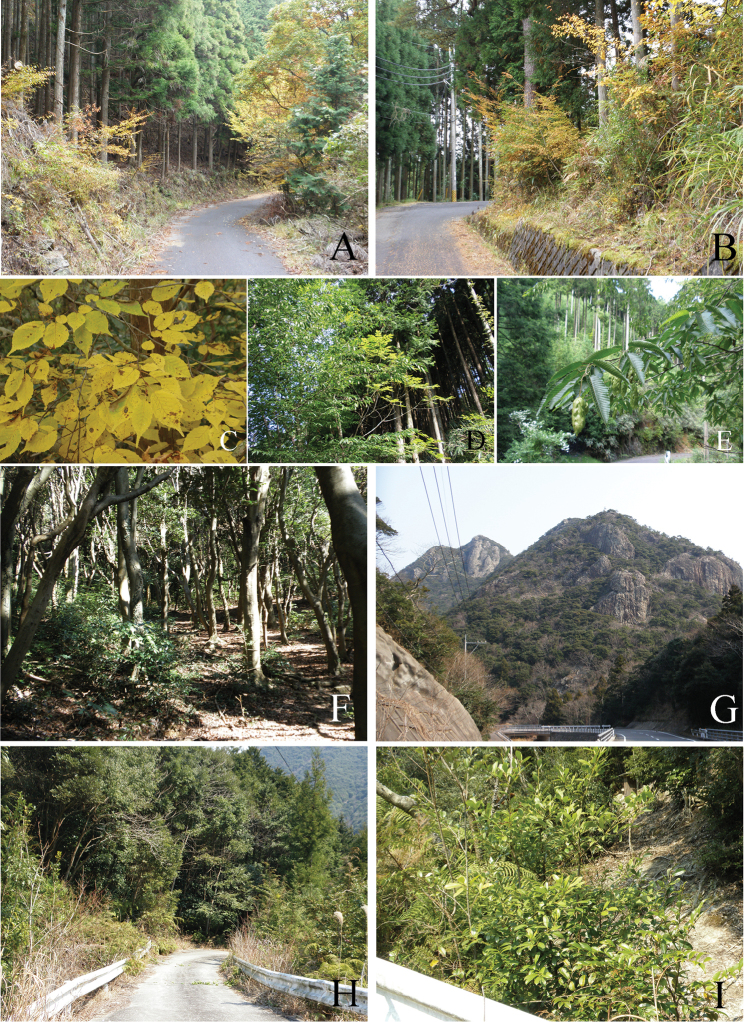
Habitats and hostplants of *Coptotriche* species. **A–E** *Coptotriche minuta* Diškus & Stonis, 2014, Soni, Nara Prefecture **F–I** *Coptotriche symplocosella* sp. n. **A** Habitat, Mitsuigatani, Konagao, 710 m **B** Habitat, Nagano, 600 m **C** Leaves of *Carpinus laxiflora* at Nagano **D** Branches of *Carpinus japonica* at Kumawata, Konagao **E** Leaves and fruits of *Carpinus japonica* at Kumawata **F** Type locality, Adachi Park, Kokura, Fukuoka Prefecture **G** Habitat, Jyozan, Mitsushima, Tsushima Is., Nagasaki Pref. **H** Habitat and host plants, *Symplocos lucida* **I** *Symplocos lucida* tree.

**Figure 2.**
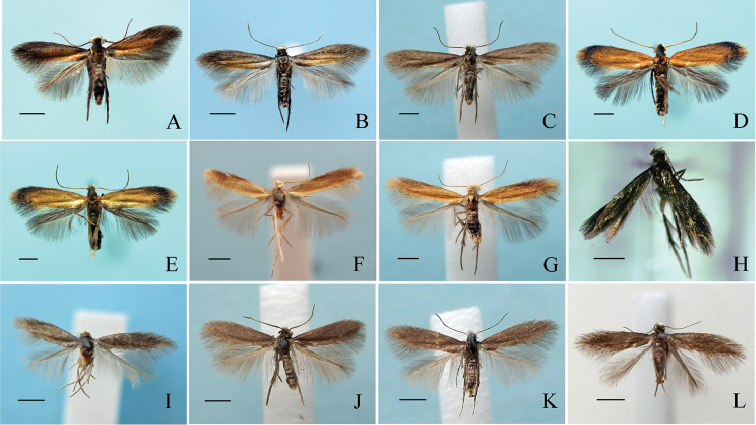
Adults of Tischeriidae species from Japan. **A** *Coptotriche minuta* Diškus & Stonis, 2014, male (Nara Prefecture) **B** Female (Nara Pref.) **C** Male, overwintering form (Nagano Pref.) **D** *Coptotriche symplocosella* sp. n., holotype male **E** Paratype female. **F** *Tischeria kumatai* sp. n., holotype male **G** Paratype female (Nagano Pref.) **H** *Tischeria relictana* Ermolaev, 1986, male (Tokushima Pref.), hostplant unknown **I** Female (Hokkaido), hostplant: *Betula ermanii* **J** Male (Nagano Pref.), hostplant: *Betula grossa* **K** Female, same hostplant **L** Female (Nara Pref.), hostplant unknown. Scale bar: 1 mm.

**Figure 3.**
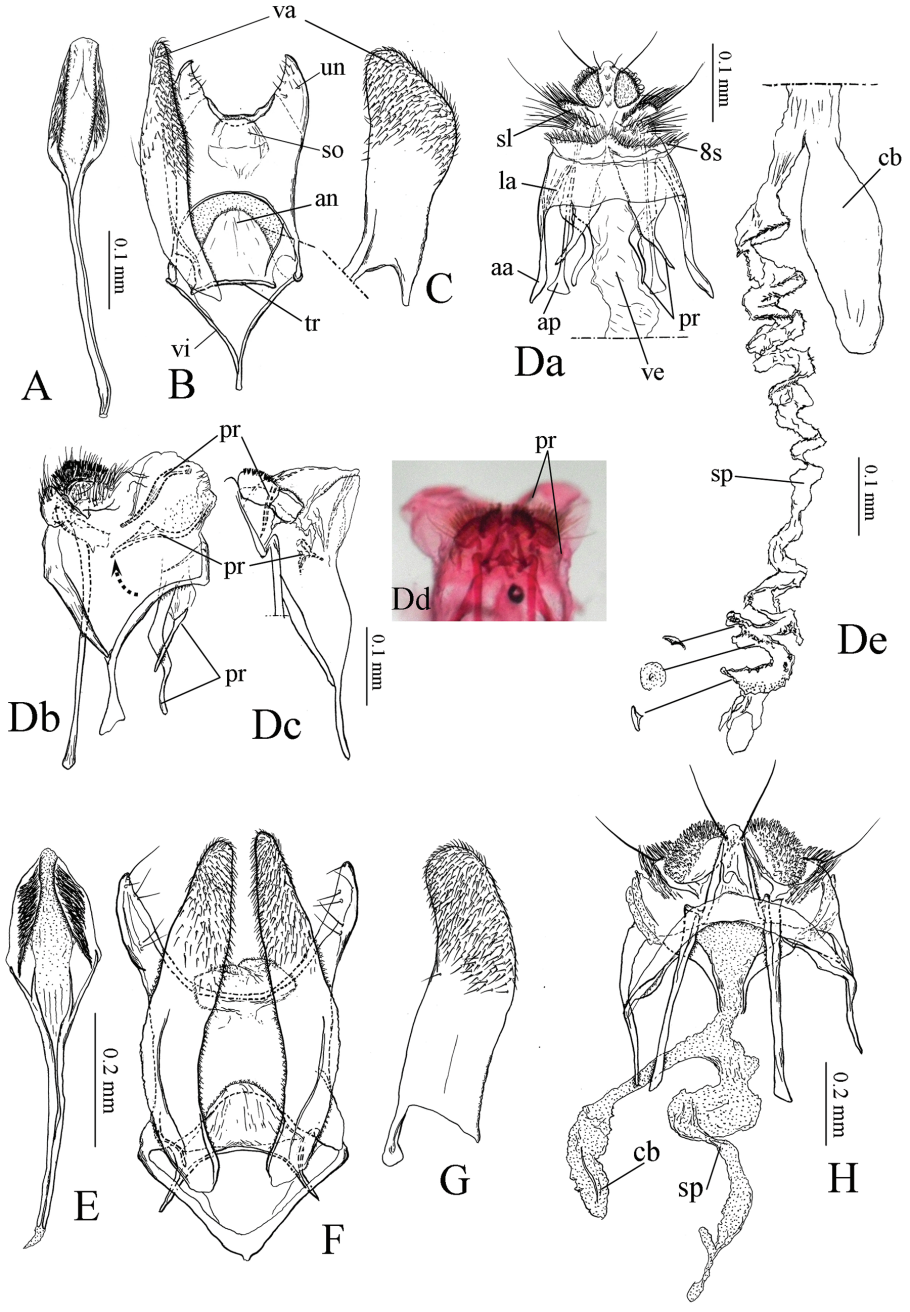
Genitalia of *Coptotriche minuta* and *Coptotriche symplocosella*. **A–D**: *Coptotriche minuta*. **E–H**: *Coptotriche symplocosella*. **A, E** Phallus, ventral view **B, F** Male genitalia, ventral view **C, G** Right valva, inner view **D, H** Female genitalia, ventral view **Db, Dc, Dd** Same, separated two pairs of prela towards posterior part **Db** Lateral view **Dcd** ventral view **De** Corpus bursae and ductus spermatheca. Abbreviations: aa: apophysis anterioris; an: anellus; ap: apophysis posterioris; cb: corpus bursae; la: lateral arm of 8th tergite; pr: prela; sl: setose lobe on 9th tergite; so: socius; sp: spermatheca; tr: transtilla; un: uncus; va: valva; vi: vinculum; ve: vestibulum; 8s: 8th sternite.

**Male genitalia** (Fig. 3A–C) (4 preparations examined).

**Female genitalia** (Fig. 3D) (3 preparations examined). Similar to *Coptotriche japoniella*, but different in having a long ductus spermathecae with convolutions; anterior part with minute spines (Fig. 3De).

#### Distribution.

Russia: the Russian Far East (Primorskiy Territory) (Stonis et al., 2014); Japan: Honshu (Nagano, Yamanashi and Nara Prefectures), Shikoku (Ehime Prefecture), Kyushu (Fukuoka Prefecture).

#### Host plants.

*Carpinus cordata* Blume, *Carpinus japonica* Blume, *Carpinus laxiflora* (Siebold & Zucc.) Blume, *Carpinus tschonoskii* Maxim., *Corylus sieboldiana* Blume and *Ostrya japonica* Sarg. (Betulaceae).

#### Biology

(Figs 7–8). The larvae were observed from June to October and hibernated in the final larval stage in Nara Prefecture. The first to second larval instars form a short linear mine towards the leaf edge (Fig. 8F, H). Later instar larvae fold the leaf edge down, forming a blotch mine, then widening it into the surrounding area (Fig. 8C, D); there are usually one to two mines per leaf (Figs 7, 8A, B, G). Frass is ejected through holes in the mine (Fig. 8H). Late and final instar larvae are about 4.0–5.5 mm long and pale yellowish green in coloration (Fig. 8E, I). The folded mines in the leaf edge are 10–20 mm in length, and the late blotch mines are 2–6 mm in width and 7–15 mm (Fig. 7B, C, E) or 20–46 mm (Fig. 7A, D) in length and ocherous in coloration. Pupation takes place within the mine.

#### Remarks.

Two pairs of prelae were observed to expand caudally and form a hump-shape in the female genitalia of some specimens (Fig. 3Db–d).

The folded, leaf-edge mines of this species resemble at first sight those of foreign congeneric species feeding on Fagaceae and Rosaceae, e.g. *Coptotriche citrinipennella* (Clemens, 1859), *Coptotriche gaunacella* (Duponchel, 1843), *Coptotriche crataegifoliae* (Braun, 1972) and *Coptotriche agrimoniella* (Braun, 1972). However, larvae of *Coptotriche citrinipennella* form more tightly folded and narrow mines (Braun 1972), while other species form more expanded mines. Fully expanded mines of *Coptotriche minuta* are easily distinguishable from those of the other Japanese *Coptotriche* species as shown in Fig. 13, although all of them are irregular blotch mines lined with a few folds. The mine of *Coptotriche minuta* is most similar to that of *Coptotriche angusticollella*, but the fold of *Coptotriche minuta* is obviously smaller than that of *Coptotriche angusticollella*. A mine of *Coptotriche minuta* may look like a pupal shelter of *Roeslerstammia pronubella* ([Denis & Schiffermüller], 1775), Roeslerstammiidae, which utilizes the same hostplant, *Carpinus laxiflora* (Hirowatari et al. 2012, figs 7, 8).

### Coptotriche symplocosella

Taxon classificationAnimaliaLepidopteraTischeriidae

Kobayashi & Hirowatari sp. n.

http://zoobank.org/C82BE915-F1FF-4FDC-8265-8C7AE249F647

Figs 2D–E , 3E–H , 9 , 10 , 11

#### Material examined.

47(11♂ 9♀ 27 exs)

#### Type material.

Holotype ♂, JAPAN: Kyushu: Adachi Park, Kokura, Fukuoka Pref., 9.iv.2012em., host *Symplocos lucida*, 20.iii.2012(larva), S. Kobayashi leg. Paratypes 10♂ 9♀, Mikata, Mitsushima, Tsushima, Nagasaki Pref., 25.iv.–6.v.2012em., S. Kobayashi leg., host *Symplocos lucida*, 27.iii.2012 (larva), SK402–405.

#### Other material.

20 exs, same data as paratypes.

Pupae. 7 exs, Mikata, Mitsushima, Tsushima, Nagasaki Pref., 27.iv.2012, S. Kobayashi leg., host *Symplocos lucida*, 27.iii.2012(larva).

#### Diagnosis.

The color of the scaling is very similar to that of many other *Coptotriche* species; the new species differs from other members of the genus in the combination of the rather long uncus (Fig. 3F), and the gently curved slender valva (Fig. 3G) in the male genitalia, and the very small corpus bursae (Fig. 3H) in the female genitalia.

**Adult** (Fig. 2D–E). Wing expanse 8.7 mm; forewing length 4.0 mm in holotype, 7.3–9.2 mm in paratypes (8.0 mm on average for 21 specimens). Head: palpi cream white to ocherous; frons smooth, blackish brown; vertex tuft blackish brown centrally, frontal and lateral tufts brown; collar brown to grayish black apically, comprised of slender lamellar scales; antenna minimally 2/3 length of forewing, brown to golden. Thorax: anterior part black, posterior part grayish black. Forewing brown to ocherous with blackish scales densely covering apex, and tipping termen and tornus; termen with brown scales. Cilia and hindwing blackish gray. Legs pale ocherous. Abdomen: black; anal tuft grayish ocherous.

**Male genitalia** (Fig. 3E–G) (2 preparations examined). Uncus with oblong claw-shaped lateral lobes. Socii membranous. Tegumen broad and rather long. Valva slender, gently curved inwards (Fig. 3G). Transtilla present. Anellus membranous, indistinct. Vinculum with rather short triangular ventral plate, with rounded anterior part. Phallus tulip-shaped, slender with broad ended apical part (Fig. 3E).

**Female genitalia** (Fig. 3H) (2 preparations examined). Similar to *Coptotriche japoniella* and *Coptotriche bifurcula*, except corpus bursae very small, slender, with two narrow signa with minute spines, and a short slender ductus spermathecae.

**Pupa.** (Fig. 11) (3 preparations examined). Brown to dark brown, 4.4–5.1 mm in length. Vertex (Fig. 11A–B) smooth, with a pair of short setae laterally (Fig. 11C). Dorsum A2–A7 with a pair of long setae, and a concentration of very small spines (Fig. 11E). Dorsum A8–A10 (Fig. 11G, H) with a pair of long dorsal spines and a pair of long lateral spines; A10 (Fig. 11F–H) furcated with a pair of short acute processes from caudal apex, rolled on the dorsal side.

#### Distribution.

Japan: Kyushu (Fukuoka and Nagasaki (Tsushima Is.) Prefectures).

#### Host plants.

*Symplocos lucida* (Thunb.) Siebold & Zucc. (Symplocaceae).

#### Etymology.

The specific epithet, *symplocosella*, refers to the genus of the hostplant, *Symplocos*.

#### Biology

(Figs 9–10). Because many young larvae were observed in leaf mines in March, the species seems to overwinter in the larval stage. The larvae mine leaves of an evergreen tree, *Symplocos lucida*, forming an elongate full-depth blotch mine beginning with a slender, linear shape (Fig. 9A, E), and gradually expanding as they feed and grow (Fig. 9B, F, G); about ~3 cm in length, white to dark yellow; the older mines turn brown in coloration (Fig. 9C). There were usually 1–3 mines per leaf (Fig. 9G). The larva ejects frass through circular holes (Fig. 10). From shed larval head capsules in the mine, we estimate that the species has six larval instars (Figs 9I, 10). The semifinal and final instar larvae are 6.0–7.0 mm long and pale green in coloration. Head capsule widths are 1st instar: 0.21 mm, 2nd: 0.25 mm, 3rd: 0.30 mm, 4th: 0.40 mm. The mature larva lines the mine with silk, so that the upper surface of the mine shows a few folds (Fig. 9C); a pupal cocoon is situated at the end of the mine.

#### Remarks.

The pupal characters of the new species are similar to those of other *Coptotriche* species, but the new species has rather short caudal processes.

### Tischeria kumatai

Taxon classificationAnimaliaLepidopteraTischeriidae

Sato, Kobayashi & Hirowatari sp. n.

http://zoobank.org/BDB99F16-F635-487C-AF81-D32A8C546829

Figs 2F–G , 4

Tischeria sp.: Sato 2011: 559

#### Material examined.

6 (3♂ 3♀)

#### Type material.

Holotype ♂, JAPAN: Hokkaido: Teine, 14.v.1959, host *Tilia japonica*, T. Kumata leg. (genitalia slide no. OPU-SK486). Paratypes Host. *Tilia japonica*: 1♂ 1♀, same locality and data of holotype, SK485; 1♀, Mt. Maruyama, Sapporo, Hokkaido, 2.v.2007em., H. Sato leg., 7.ix.2006(larva); 1♀, Oshirakawa, Azumi, Nagano Pref., 31.v.1992em., N. Hirano leg., 7.ix.1991, SK567. Host *Tilia* sp.: 1♂, Mt. Maruyama, Sapporo, Hokkaido, 24.iii.2007em., H. Sato leg., 7.ix.2006(larva).

#### Diagnosis.

**Figure 4.**
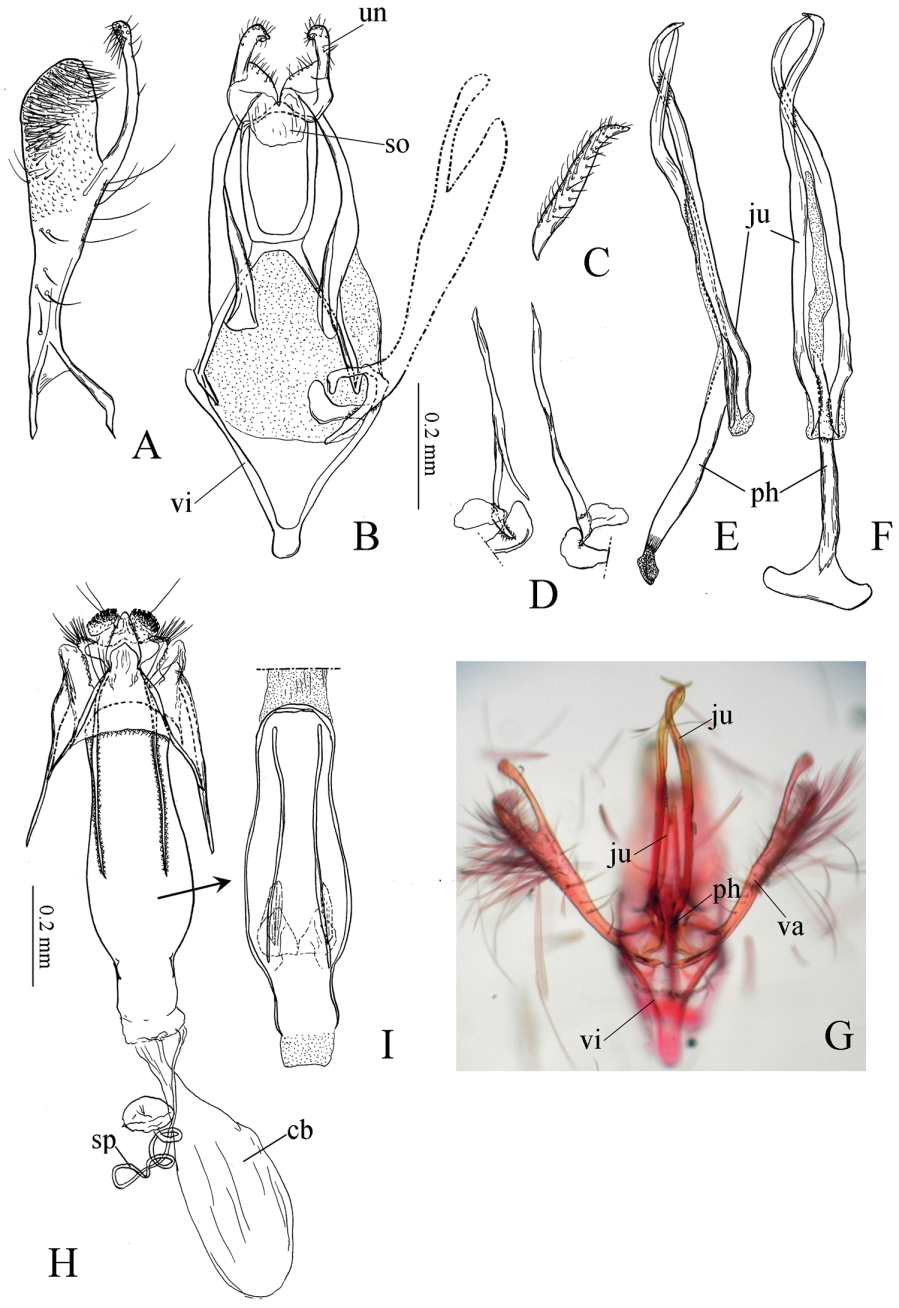
Genitalia of *Tischeria kumatai*. **A** Left valva, inner view **B** Male genitalia with phallus, juxta and left valva removed, ventral view **C** Left lateral lobe of uncus, lateral view **D** Juxta, ventral view **E** Phallus and juxta, lateral view **F** Same, ventral view **G** Valvae, phallus, juxta and vinculum, ventral view **H** Female genitalia, distal part, ventral view **I** Antrum, ventral view **J** Corpus bursae, ventral view. Abbreviations: ju: juxta; ph: phallus; so: socius; un: uncus; va: valva; vi: vinculum.

The color of the scaling of this new species has little or no diagnostic value. However, the female genitalia exhibit good diagnostic characters, especially the thickened plate-like vestibulum (antrum) (Fig. 4I). Among *Tischeria* species having a similarly unusual antrum (e.g. *Tischeria ptarmica* Meyrick (see van Nieukerken 2010) and *Tischeria zestica* Meyrick (see Puplesis and Diškus 2003), the new species is most similar to *Tischeria unca* Diškus & Stonis from the Russian Far East (feeding on *Quercus*), but is recognizable by the slender posterior plate of the antrum and the lack of spines in the corpus bursae in the female genitalia, and the long spiral shaped juxta (Fig. 4E–F) and the valva with a very slender basal half (Fig. 4A) in the male genitalia. The South African species, *Tischeria antilope* Puplesis, Diškus & Mey (female unknown) also has a similarly shaped juxta and valva, but differs from the new species by the narrow ventral plate of the vinculum, the longer valva and the lack of a pair of short lateral processes on the juxta (Puplesis and Diškus 2003, figs 586, 589). A Far Eastern Russian species, *Tischeria puplesisi* Kozlov (female unknown), differs from the new species by the broader valva and short, stout juxta (Kozlov 1986, fig. 2).

**Adult** (Fig. 2F–G). Wing expanse 7.3 mm; forewing length 3.5 mm in holotype, 7.4 and 8.4 mm (3.3 and 3.9 mm) in paratypes. Head: palpi cream white; face smooth, cream white; vertex tuft white mixed with slender pale ocherous lamellar scales; antenna slightly longer than or equal to half of forewing, basally pale ocherous, apically ocherous to brown. Thorax pale ocherous. Forewing pale ocherous with scattered brown scales on the costal half towards the apical area. Cilia and hindwing blackish gray. Legs white to pale ocherous. Abdomen: brown; anal tuft grayish ocherous.

**Male genitalia** (Fig. 4A–G) (1 preparation examined). Uncus with very slender lateral lobes (Fig. 4C). Socii membranous. Tegumen narrow, marginally reinforced with a pair of slightly inwardly curved arms (Fig. 4B). Valva long, basally very slender, apically rounded, distally covered with fine setae and with a long slender dorsal process (Fig. 4A). Ventral plate of vinculum long and narrow, rounded anteriorly. Juxta very long, comprising two pairs of spiral curved processes (Fig. 4D–F); one pair connecting to the middle of the phallus, equal to the length of valva, apically forming a spiral shape (Fig. 4E–F); the other pair more slender, half the length of the valva, basally slightly curved laterally (Fig. 4D). Phallus slender, distinctly broadened at basal end, forming a pale slender membranous structure from the middle to apex.

**Female genitalia** (Fig. 4H–I) (2 preparations examined). Antrum (Fig. 4I) strongly sclerotized, plate-like, slightly broadened medially, with a pair of pale spatulate plates at 1/3 of antrum and a pair of very slender long processes. Ductus spermathecae membranous and slender, with 3–4 coils. Corpus bursae small and smooth (Fig. 4H).

#### Distribution.

Japan: Hokkaido, Honshu (Nagano Prefecture).

#### Host plants.

*Tilia japonica* (Miq.) Simonk. (Malvaceae).

#### Etymology.

The specific epithet, *kumatai*, is dedicated to Dr Tosio Kumata, who is one of the great Lepidoptera taxonomists and collected the holotype and some of the paratypes.

#### Biology.

The larvae form dark gray blotch mines on the leaf edge which are very similar to the folded mines of *Coptotriche minuta* on *Carpinus japonica*. *Tischeria kumatai* is common in Nagano Prefecture.

### Tischeria relictana

Taxon classificationAnimaliaLepidopteraTischeriidae

Ermolaev, 1986

Figs 2H–L , 5

Tischeria relictana Ermolaev, 1986: 6–8, fig. 1
Tischeria sp.: Sato 2011: 127, 559, fig. II-14.2H.
Tischeria sp.: Hirowatari et al. 2015: 26.

#### Type locality.

Russia: the Russian Far East (Sakhalin).

#### Material examined.

6(3♂ 3♀)

Host *Betula ermanii*: 1♂ 1♀, Sapporo, Hokkaido, 1.v.1959, T. Kumata leg., HK387♂, SK487♀.

Host *Betula grossa*, N. Hirano leg.: 1♂, Oshirakawa, Azumi, Matsumoto, Nagano Pref., 25.iv.1990em., 23.x.1989(larva), SK565; 1♀, same locality, 20.v.2004em., 11.x.2003(larva), SK566.

Host unknown: 1♀, Mt. Wasamata, Nishihara, Kamikitayama, Nara Pref., 23.viii.2011(L.T.), T. Hirowatari, K. Ikeuchi, Y.-S. Bae & S. Kobayashi leg., SK569. 1♂, Kumosa-yama, Kamiyama, Tokushima Pref., 22.viii.2010(L.T.), K. Yamada, T. Hirowatari, K. Ikeuchi, S. Kobayashi and K. Akita leg., SK463, deposited in TKPM.

#### Diagnosis.

**Figure 5.**
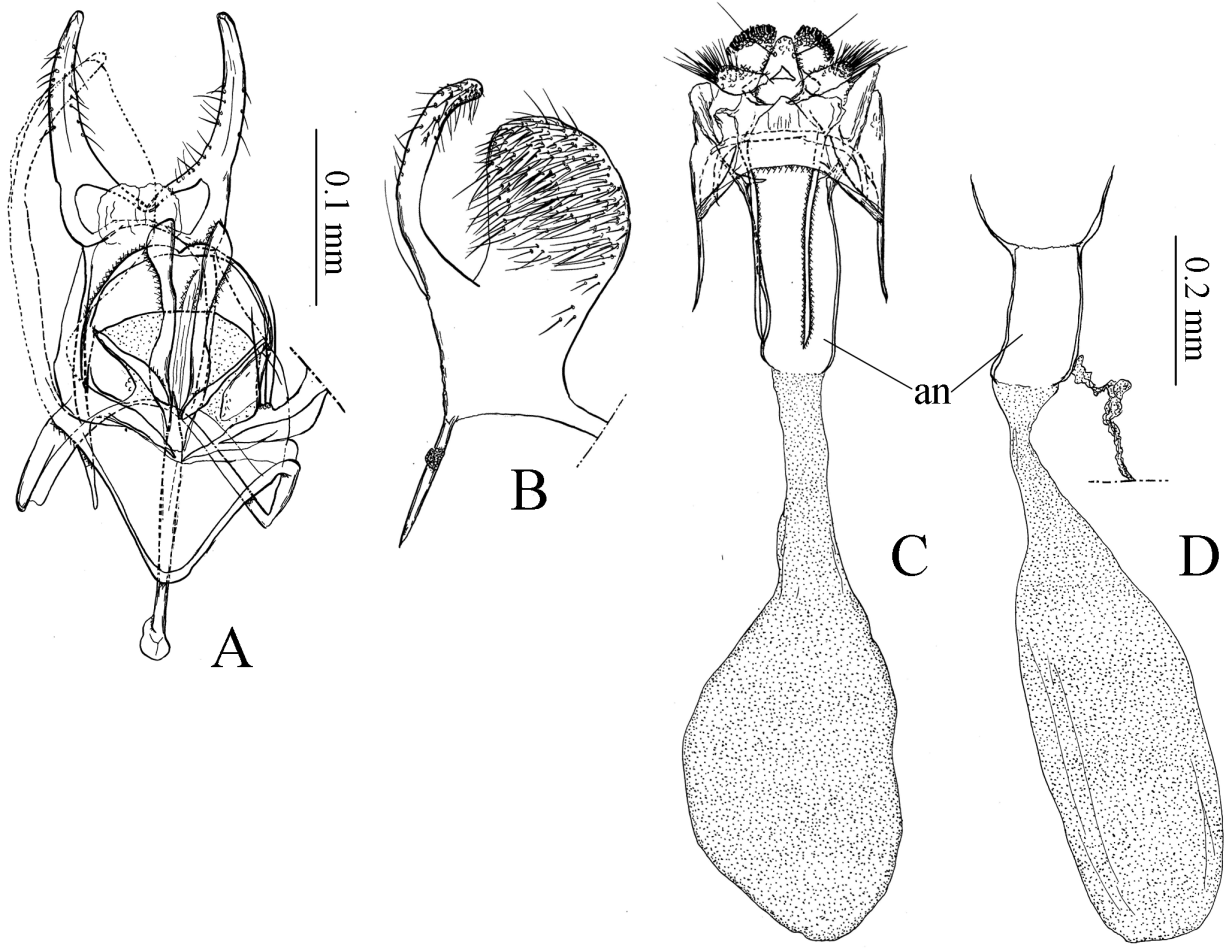
Genitalia of *Tischeria relictana*. **A** Male genitalia, ventral view, with right valva removed **B** Right valva, inner view **C** Female genitalia, ventral view (hostplants: *Betula grossa*) **D** Antrum, spermatheca and corpus bursae (hostplants unknown). Abbreviations: an: antrum.

*Tischeria relictana* resembles *Coptotriche* species associated with Rosaceae in that the wings and thorax are covered with gray scales. However, this species can be regarded as a member of *Tischeria* by the presence of a developed juxta in the male genitalia. Although having divided valvae as well as some other congeneric species (e.g., *Tischeria zestica* Meyrick and *Tischeria martinkrugeri* Puplesis & Diškus), *Tischeria relictana* clearly differs from the others in the double juxta with anteriorly semicircular sclerotized diaphragma (Fig. 5A). A Far Eastern Russian species, *Tischeria sichotensis* Ermolaev, has female genitalia similar in shape to *Tischeria relictana*, but the former is separated from the latter by the presence of two acute lateral lobes of the antrum and the short spine-like pectinations in the caudal part of the corpus bursae (Stonis et al. 2014, figs 42, 43).

#### Additional description.

**Adult** (Fig. 2H–L). Male and female. Wing expanse 6.1–7.1 mm; forewing length 3.0–3.2 mm in Japanese specimens. Head: palpi blackish brown; face smooth, blackish brown; vertex tuft blackish brown; antenna blackish brown, equal to half of forewing in length. Thorax black. Forewing blackish brown to black. Cilia and hindwing blackish gray. Legs blackish brown. Abdomen blackish brown; anal tuft grayish ocherous.

**Male genitalia** (Fig. 5A, B) (2 preparations examined). Uncus with long and very slender lateral lobes. Socii membranous. Tegumen strongly sclerotized marginally with a pair of slightly inwardly curved frames. Valva (Fig. 5B) broad, covered distally with fine setae, and having a long, slender, dorsal process. Ventral plate of vinculum narrow, triangular. Juxta short, comprising two pairs of processes, one pair connecting to the middle of the phallus, half the length of the valva, narrow medially, broadened apically; the other pair needle-shaped, 1/4 length of valva, slightly broadened basally. Transtilla absent. Diaphragma anteriorly sclerotized, a semicircular plate, folding round the phallus and contacting the needle-shaped part of the juxta ventrally. Phallus (Fig. 5A) slender, distinctly broadened at basal end, forming a pale slender membranous structure from the middle to apex.

**Female genitalia** (Fig. 5C, D) (3 preparations examined). Similar to *Tischeria sparmanniae* and *Tischeria zestica*, but differs in having short apophyses anteriores and posteriores, a slender ductus bursae and the corpus bursae without spines.

#### Distribution.

Russia: the Russian Far East (Sakhalin) (Ermolaev 1986); Japan: Hokkaido, Honshu (Nagano and Nara Prefectures), Shikoku (Tokushima Prefecture).

#### Host plants.

*Betula ermanii* Cham., *Betula grossa* Siebold & Zucc. (Betulaceae).

#### Biology.

The detailed biology of this species is unknown. The larvae mine leaves of *Betula* spp., according to label data of adult specimens.

#### Remarks.

We collected a female adult of this species at a light trap on Mt. Wasamata, Nara Prefecture, where we also collected tischeriid mines on *Betula grossa* (Fig. 12). The larvae formed an ocherous to dark gray oblong mine, similar to that of *Coptotriche minuta*, on the leaf edge or along the leaf vein. Unfortunately, adults did not emerge. We also collected a male adult of this species in a light trap in a deciduous broadleaf forest where *Betula grossa* grows, on Mt. Kumoso, Tokushima Prefecture. C. Doorenweerd (pers. comm. E.J. van Nieukerken) collected a pupa in Hokkaido from a folded leafmine at the leaf edge on *Betula* of which the DNA barcode groups with other species of *Tischeria* and not *Coptotriche*. Judging from these data, the mines on *Betula* can be considered to have been made by *Tischeria relictana*.

### Tischeria decidua siorkionla

Taxon classificationAnimaliaLepidopteraTischeriidae

Kozlov, 1986

Fig. 6

Tischeria decidua Wocke, 1876: 41–43; Sato 1993: 552–553, fig. 4.
Tischeria decidua siorkionla Kozlov, 1986: 25; Stonis et al. 2014: 148–151, figs 25–35.

#### Type locality.

Russia: the Russian Far East (Primorskiy Territory).

#### Material examined.

29(18♂ 11♀)

Host *Quercus acutissima*: 2♂ 3♀, Komaga-take-SA, Komagane, Nagano Pref., 12–15.viii.2010em., S. Kobayashi leg., 1.viii.2010(larva), SK554, 594; 9♂ 8♀, Tsubata-cho, Ishikawa Pref., ix.1983em., I. Togashi leg., viii.1983, HK705, 706; 1♂, Awara-cho, Fukui Pref., 25.viii.1987em., HK948; 1♂, Kazura, Soni, Uda, Nara Pref., 31.vii.2013em., S. Kobayashi leg., 13.vii.2013(larva), SK555; 2♂, Iwawaki-san, Osaka Pref., 23.v.1980em., H. Kuroko leg., 3.xi.1979.

Host *Quercus crispula*: 2♂, Minodo, Nagano Pref., 13.v.1980em., H. Kuroko leg., SK595.

Host unknown: 1♂, Ohdaigahara, Kamikitayama, Yoshino, Nara Pref., 20.vii.2009(L.T.), T. Hirowatari, K. Ikeuchi, S. Kobayashi, K. Akita, A. Inotsuka & T. Yoshida leg., SK214.

#### Diagnosis.

See Stonis et al. (2014).

**Figure 6.**
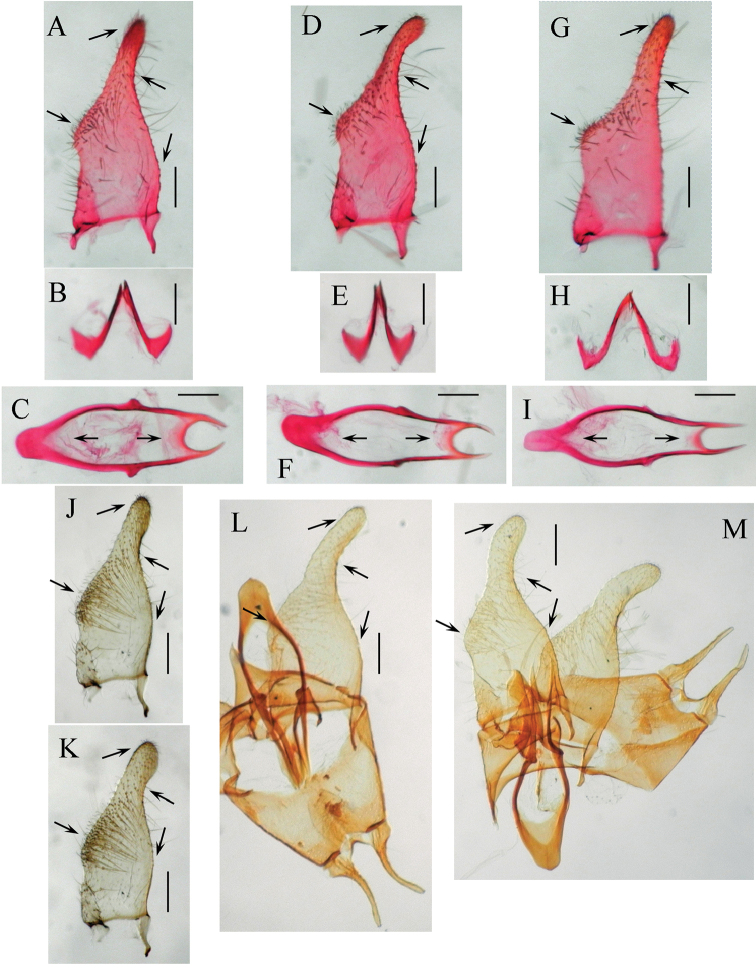
Male genitalia of Japanese specimens of *Tischeria decidua siorkionla*. **A, D, G, J, K** Right valva, outer view **B, E, H** Juxta, ventral view **C, F, I** Phallus, ventral view **L, M** Preparation by Dr H. Kuroko **A–F, J, L** Host *Quercus acutissima* **G–I** Host *Quercus crispula* **K** Host unknown **A–C** Osaka Pref., genitalia slide no. SK596 **D–F** Nagano Pref., SK594 **G–I** Nagano Pref., SK595 **J** Nara Pref., SK555 **K** Nara Pref., SK214 **L** Ishikawa Pref., HK705 **M** Same data, HK706. Scale bar 100 μm.

**Male genitalia** (Fig. 6). (9 preparations examined). See Sato (1993, 4A–E) and Stonis et al. (2014, fig. 26–29, 31, 33, 35).

**Female genitalia** See Sato (1993, fig. 4F).

#### Distribution.

Russia: the Russian Far East (Primorskiy Territory); Japan: Hokkaido, Honshu, Kyushu (Tsushima Is.).

#### Host plants.

*Quercus acutissima* Carruth., *Quercus crispula* Blume, *Quercus dentata* Thunb., *Quercus serrata* Thunb., and *Quercus variabilis* Blume, Fagaceae in Japan (Sato 1993). *Quercus mongolica* Fisch. ex Ledeb. and *Quercus serrata* in Russia (Kozlov 1986; Stonis et al. 2014).

#### Biology.

(Fig. 13–3). See Sato (1993) and Stonis et al. (2014).

#### Remarks.

In Japan, this species had been treated as ‘*Tischeria decidua* Wocke’ until the East Asiatic subspecies *Tischeria decidua siorkionla* was described by Kozlov (1986). Stonis et al. (2014) reported that Japanese representatives of *Tischeria decidua* belonged to the subspecies *siorkionla* Kozlov. In the Japanese specimens we studied, the apex of the valva is broader (present study: 50–65 μm; Stonis et al. (2014): 65 μm), but other characters were considered to lie within the range of individual variation, e.g., some specimens have a rather prominent median bulge and sinuous inner margin of the valva (Fig. 6A, D, G, L; Sato 1993, 4D), i.e. more similar to that of the nominotypical European subspecies; others have a rather longer but less prominent median bulge and nearly straight inner margin of the valva (Fig. 6J, K, M; Sato 1993, fig. 4A, E), i.e. more similar to that of *Tischeria decidua siorkionla*. The chitinized basal part of the phallus tends to be less developed and the transverse bar is shorter in Japanese material than in the nominotypical European subspecies (Fig. 6C, F, I), as shown by Stonis et al. (2014). In conclusion, we treat the Japanese representatives as *Tischeria decidua siorkionla* following Stonis et al. (2014) on the basis of the broader apex of the valva and the less developed basal part of the phallus (Fig. 6).

## Discussion

In the present study, a total of eleven Tischeriidae species are recognized from Japan, not including an unidentified *Tischeria* species which occurs on evergreen *Quercus* (recorded by Sato 2011), of which nine of them were reared by us. It is revealed that the previously unknown hostplants for *Coptotriche minuta* were *Carpinus* spp., *Corylus sieboldiana* and *Ostrya japonica*, Betulaceae, while those for *Tischeria relictana* were *Betula* spp, Betulaceae. *Coptotriche symplocosella* and *Coptotriche japoniella* utilize evergreen plants of Ericales as hosts and hibernate in the larval stage. Oishi and Sato (2009) reviewed the voltinism and leaf type of hostplants of the Tischeriidae; *Coptotriche japoniella* has a univoltine life cycle and long larval period, and overwinters as 5^th^ instar. The seasonal development of *Coptotriche symplocosella* was not examined, but it has a similar hibernating form and larval mine as *Coptotriche japoniella*. Other Japanese species including *Tischeria kumatai* and *Coptotriche minuta* probably have a bivoltine life cycle and hibernate as mature larva within the cocoon. The overwintering generation of *Coptotriche minuta* has color morphs of forewings from ocherous to black and a larger body size than other generations. According to Kuroko (1982), *Coptotriche szoecsi japonica* has a brighter color and smaller body size compared with the nominotypical subspecies *szoecsi*. The subspecies *japonica* was described from specimens collected in May and July. The polytypic concept of *Coptotriche szoecsi* could not be confirmed, because there existed no opportunity to examine additional material.

Figures 12 and 13 provide a pictorial key to the leafmines of Japanese Tischeriidae. Mines of *Coptotriche* species are distinguished from those of blotch miners of other lepidopteran families by the ejection of frass through holes and the folds of the fully expanded mine; e.g., *Coptotriche japoniella* (Fig. 13(8ab)) is distinguished by these characters from the *Eurya* blotch miner, *Lyonetia euryella* Kuroko, 1964. According to Sato (1993), Japanese *Tischeria* mines are distinguished mainly by colors and patterns of the surface and cocoon nidus (Fig. 13(1–3)).

As regards the morphology of the female genitalia, Puplesis and Diškus (2003) pointed out that the corpus bursae and ductus spermathecae have great value in generic and species diagnoses. *Coptotriche* species were often distinguished from one another by the size of the corpus bursae and the length of the ductus spermathecae. Virgin female adults of five Japanese *Coptotriche* species obtained from rearing in the laboratory were examined, not including *Coptotriche szoecsi* (Kasy). The size of the corpus bursae and the length of the ductus spermathecae differed from one another as indicated in the species descriptions, e.g., the corpus bursae of *Coptotriche angusticollella* was larger than in the other four species.

**Figure 7.**
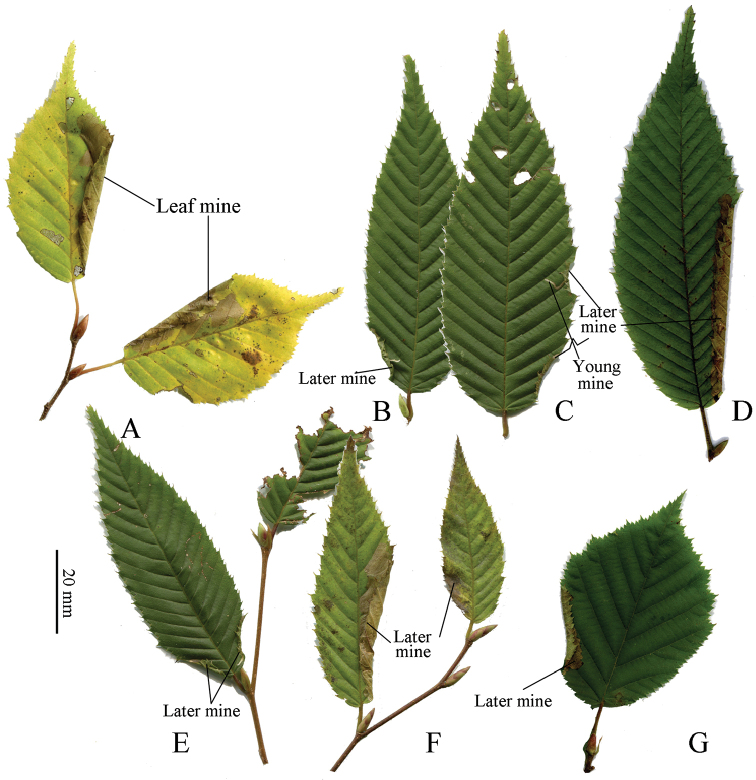
Mines of *Coptotriche minuta* on its hostplants. **A** *Carpinus laxiflora* **B–F** *Carpinus japonica* **G** *Corylus sieboldiana*.

**Figure 8.**
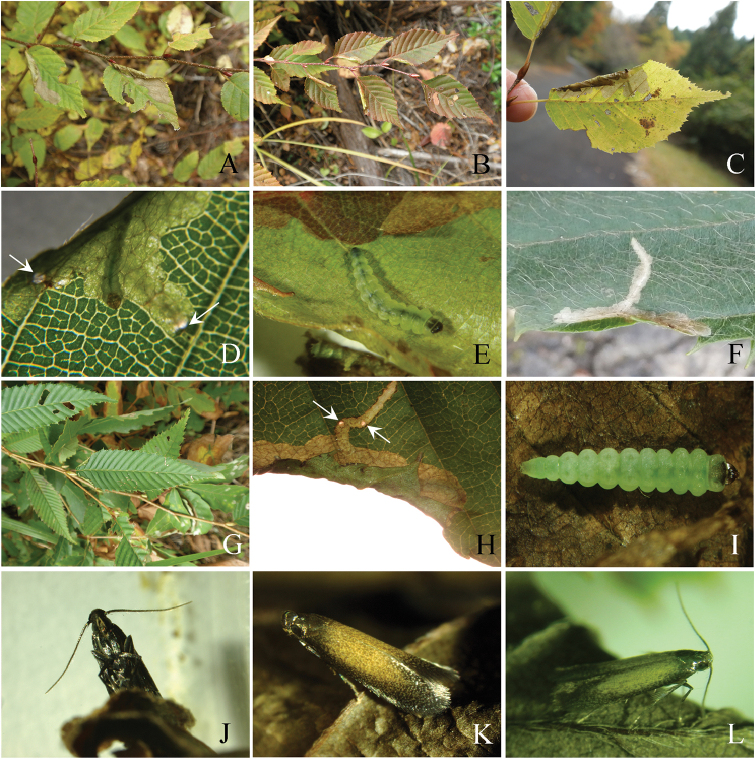
Biology of *Coptotriche minuta* and its hostplants. **A–E** *Carpinus laxiflora* **F** *Carpinus tschonoskii* **G–J** *Carpinus japonica* **L** *Corylus sieboldiana* **A–C, G** Blotch mines and branches of hostplant **D** Mine by later instar larva **E** Later instar larva **F, H** Young mines **I** Later instar larva in winter **J** Resting posture of the adult, ventral view **K** Same, dorsal view **L** Same, lateral view. Arrows show holes for ejecting frass.

**Figure 9.**
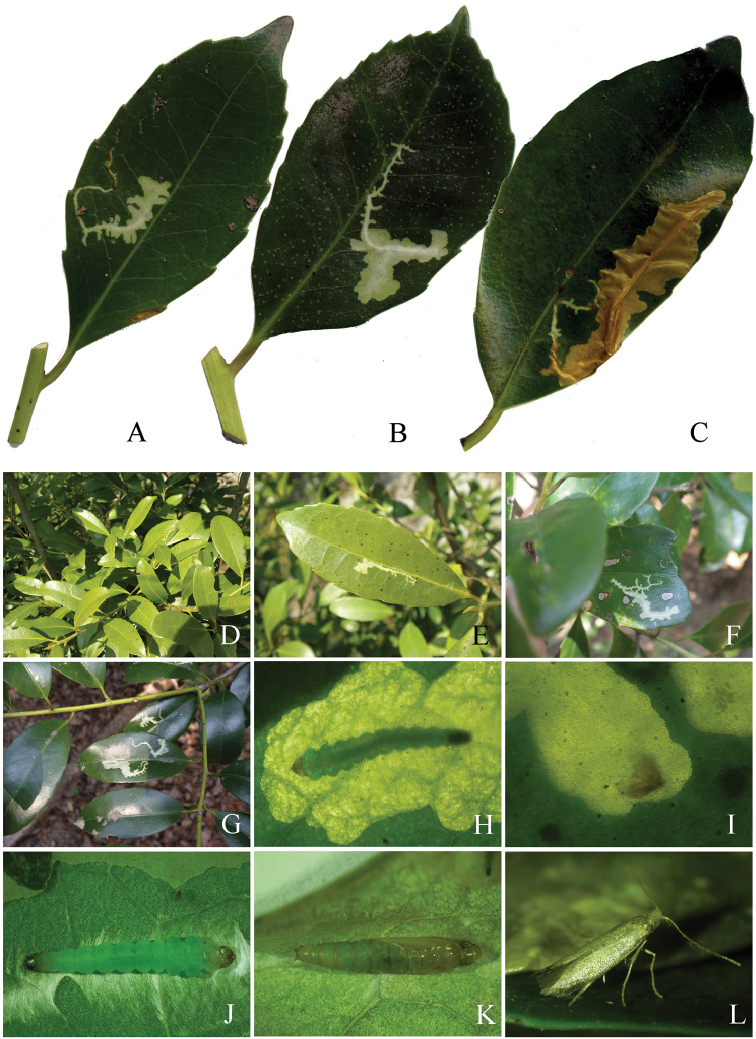
Biology of *Coptotriche symplocosella* and its hostplant, *Symplocos lucida*. **A, E** Young mines **B, F–G** Later mines **C** Old mine **D** Young mines and shoots of *Symplocos lucida* **H** Later instar larva **I** Head capsule within mine **J** Final instar larva, dorsal view **K** Pupa, dorsal view **L** Resting posture of adult, lateral view.

**Figure 10.**
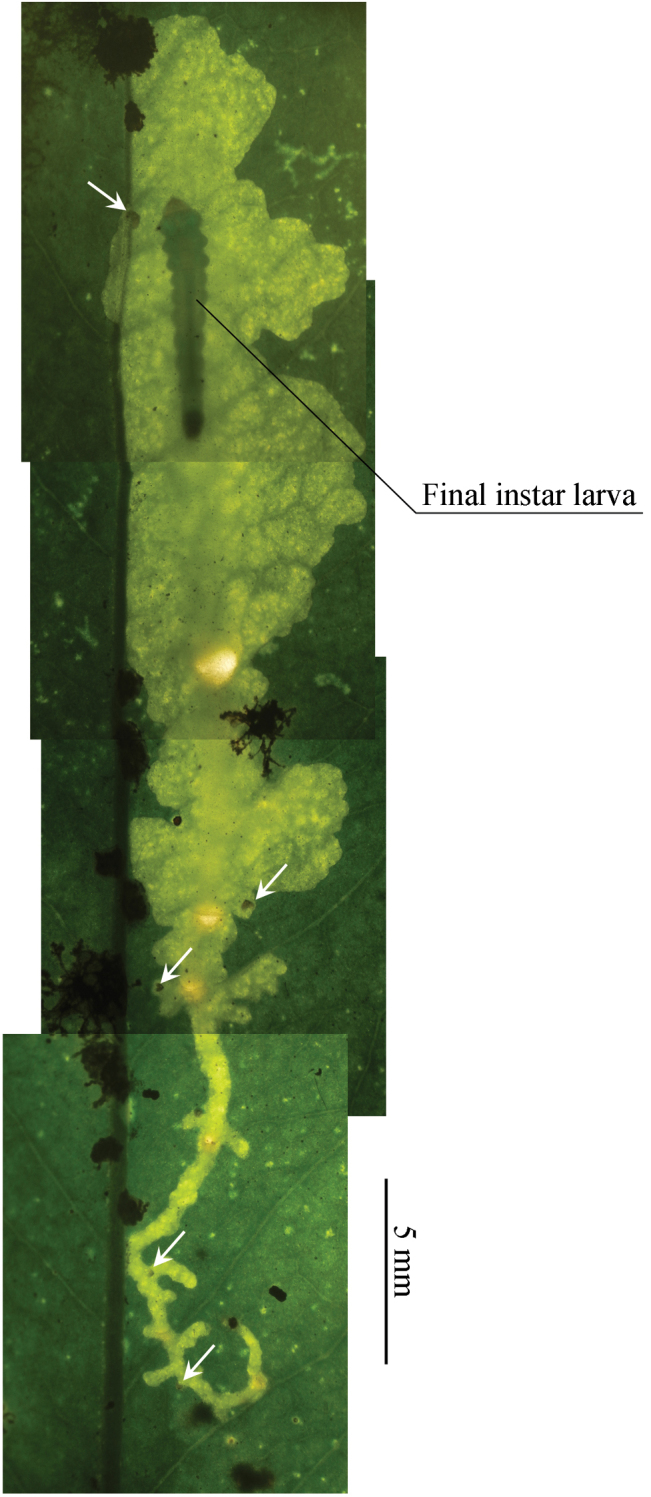
Immature stage of *Coptotriche symplocosella* and its hostplant, *Symplocos lucida*. Arrows show head capsules.

**Figure 11.**
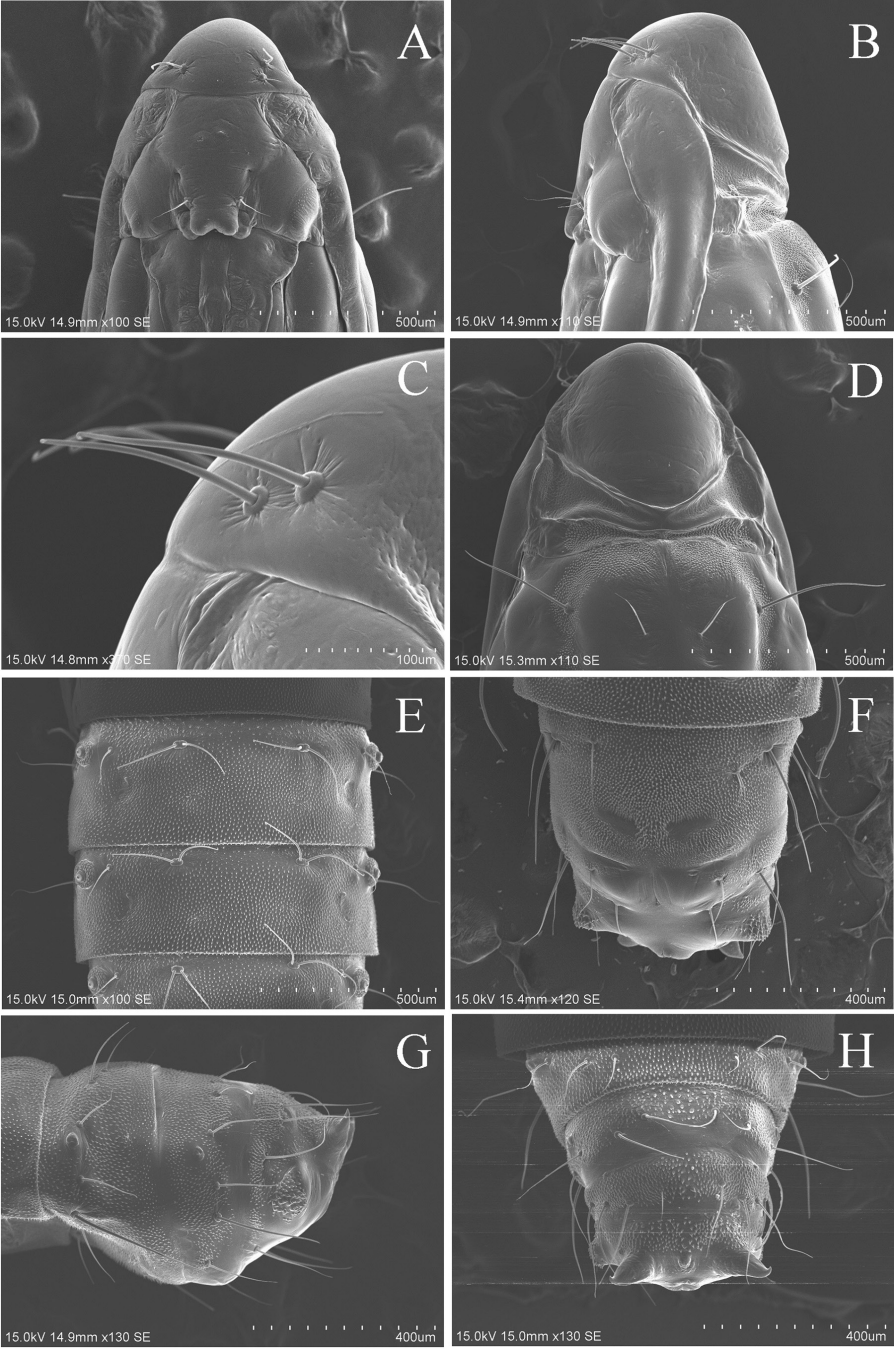
Pupa of *Coptotriche symplocosella*. **A** Head, ventral view **B** Lateral view **C** Frontal setae, lateral view **D** Head, dorsal view **E** Setae of abdominal tergum **F** A8–A10, ventral view **G** Lateral view **H** Dorsal view.

**Figure 12.**
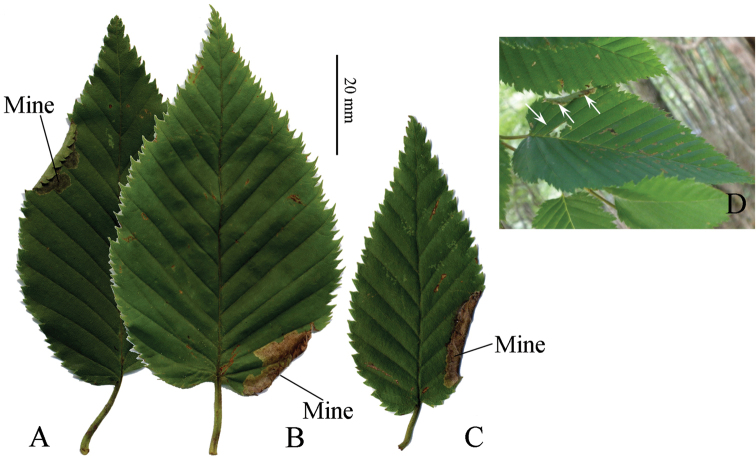
Mines of Tischeriidae species, possibly *Tischeria relictana*, and its hostplant, *Betula grossa*. **A–C** Folded mines **D** Folded mine and blotch mine among leaf veins. See remarks of *Tischeria relictana*.

**Figure 13.**
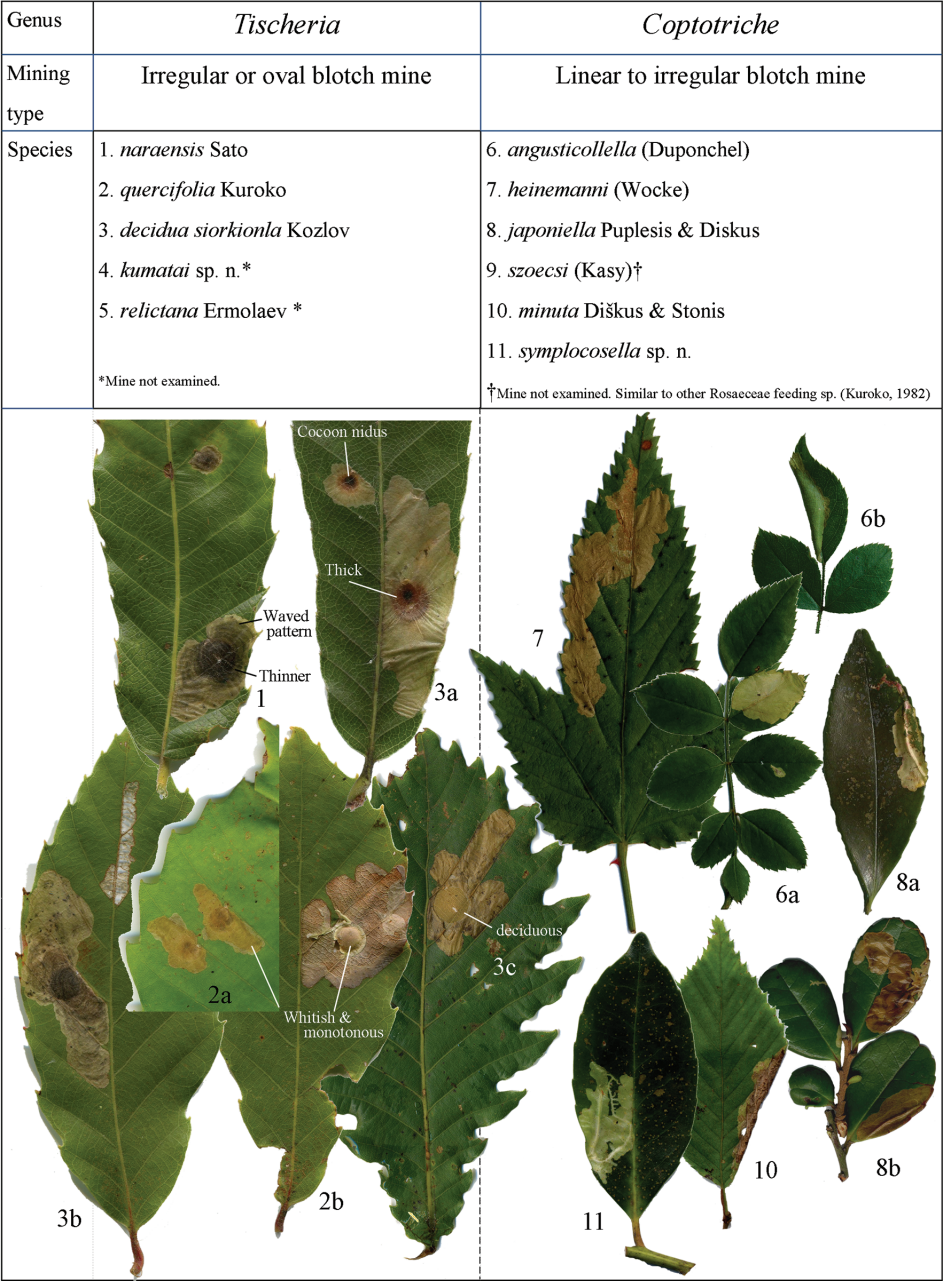
Mine characters of Japanese Tischeriidae. Hostplants: **1, 3a** *Quercus acutissima* **2, 3b** *Quercus serrata* **3c** *Quercus crispula* **6** *Rosa multiflora* **7** Rubus palmatus var. palmatus **8a** *Eurya japonica* **8b** *Eurya emarginata* **10** *Carpinus laxiflora* **11** *Symplocos lucida*. Mine characters of *Tischeria* species follow Sato (1993).

## Supplementary Material

XML Treatment for Coptotriche minuta

XML Treatment for Coptotriche symplocosella

XML Treatment for Tischeria kumatai

XML Treatment for Tischeria relictana

XML Treatment for Tischeria decidua siorkionla
